# MRI compatible detector design: experience from the development of a digital SiPM based detector stack for simultaneous PET/MRI

**DOI:** 10.1186/2197-7364-1-S1-A17

**Published:** 2014-07-29

**Authors:** Peter Michael Dueppenbecker, Bjoern Weissler, Pierre Gebhardt, Jakob Wehner, David Schug, Volkmar Schulz

**Affiliations:** Department of Physics of Molecular Imaging Systems, Institute for Experimental Molecular Imaging, RWTH Aachen University, Aachen, Germany; Division of Imaging Sciences and Biomedical Engineering, King’s College London, London, UK; Philips Research Laboratories, Aachen, Molecular Imaging Systems, Kragujevac, Germany

Silicon based photon detectors make it nowadays possible to build highly integrated PET detectors for simultaneous PET/MRI. Although the operating principle of silicon photomultipliers is believed to be insensitive to the strong magnetic fields inside MRI machines, the construction of an MRI compatible detector has to cover considerably more aspects of MRI compatibility. In this paper we report on our development of an MRI compatible PET detector stack based on digital SiPMs for the Hyperion IID scanner.

We developed an MRI compatible PET detector stack composed of two main assembly groups, called sensor tile and interface board. The sensor tile contains an array of 64 digital SiPM and is connected via two connector to the interface board. The interface board contains a Xilinx Spartan 6 FPGA for data collection, configuration and voltage control.

We investigated the PET performance and MRI compatibility of the detector stack in combination with pixilated LYSO scintillator arrays with 1 mm pitch and 4 mm pitch inside a 3 Tesla Philips Achieva MRI system. We used MRI sequences with intensive gradient switching, field homogeneity and spurious noise scans to investigate PET/MRI interference effects. Additionally we build up laboratory setups with electromagnets and strong permanent magnets to study gradient induced effects and B0 dependencies in more detail.

The detector stack is fully functional inside the B0 field and position histograms are undistorted. Energy histograms showed on average a 1 % upward shift of the 511 keV photopeak position caused by a shift of bias voltage supply. Initially observed effects of gradient switching on energy resolution and stability could be traced back to noise pickup of the voltage controllers.

We couldn't observe any direct effect of the MRI environment on the digital SiPM itself. In fact MRI compatibility in practice is determined by proper design of the entire system.

